# Effectiveness of Cleaning the Laparoscopic Lens With a Novel Microfiber Cloth During Laparoscopic Colorectal Cancer Surgery: A Prospective Randomized Controlled Trial

**DOI:** 10.1111/ases.70124

**Published:** 2025-08-03

**Authors:** Kiyoaki Sugiura, Koji Okabayashi, Ken Yamataka, Ryo Seishima, Kohei Shigeta, Masashi Tsuruta, Yuko Kitagawa

**Affiliations:** ^1^ Department of Surgery Eiju General Hospital Tokyo Japan; ^2^ Department of Surgery Keio University School of Medicine Tokyo Japan; ^3^ Department of Hepato‐Biliary‐Pancreatic & Gastrointestinal Surgery International University of Health and Welfare, School of Medicine Chiba Japan

**Keywords:** colorectal cancer, laparoscopic lens fogging, laparoscopic surgery, Toraysee

## Abstract

**Introduction:**

In this randomized controlled trial, we compared the effectiveness of Toraysee for ES (Toray, Tokyo, Japan), a microfiber cleaning cloth composed of ultrafine polyester fibers, with that of a conventional lens‐cleaning method during laparoscopic colorectal surgery.

**Methods:**

Patients scheduled for laparoscopic colorectal surgery were randomly allocated to a microfiber cleaning cloth or a conventional lens‐cleaning arm. The primary outcome was the mean time spent on each occasion of lens cleaning. Visual quality was evaluated objectively by using visual analyzing software.

**Results:**

Sixty patients consented to participation in this study, and the data of 55 patients were analyzed. The mean time spent on each occasion of lens cleaning was significantly shorter in the microfiber cloth group than in the control group (16.12 ± 4.63 vs. 21.04 ± 4.83, *p* < 0.01). Despite the shorter cleaning time, the median difference in clarity score between before and after each cleaning occasion did not differ significantly between the groups (0.164 vs. 0.135, *p* = 0.276).

**Conclusions:**

Toraysee microfiber cleaning cloths are easy and time efficient lens‐cleaning devices while achieving a visual quality equivalent compared to a conventional cleaning method.

## 1 Introduction

Today, laparoscopic surgery is widely accepted and has become a standard surgical modality for many surgeons. Laparoscopic surgery depends heavily on the laparoscope and the vision it provides. Technological advances in surgical optics have been remarkable, enabling surgeons to perform surgeries with 3D vision or 4K resolution. This facilitates faster surgeries without compromising safety and with no adverse effects on the surgeon. The European Association of Endoscopic Surgery reached a consensus that the use of 3D technology is associated with both the shortening of the operative time and a significant reduction in complication rates, especially for surgeries that involve laparoscopic suturing [1]. Mari et al. have reported a cohort study of CRC surgery that showed that 4K technology is associated with reduced intraoperative blood loss and operative time [2]. A clear laparoscopic view is a prerequisite for benefiting from these technological advances. Laparoscopic lens fogging (LLF) can be caused by an imbalance of temperature between the scope lens and abdominal cavity and by particulate debris, blood, and smoke accumulation on the scope lens. These factors have been problematic since the introduction of laparoscopic surgery and remain unsolved thus far.

Many techniques for reducing the adverse effect of lens contamination have been developed. Because LLF has largely been attributed to temperature and humidity changes during laparoscopic surgeries, [3] surfactants have been widely applied in an attempt to minimize this problem. Those most frequently used in Japan are Ultra‐Stop (Sigmapharm, Vienna, Austria), Dr. Fog (Aspen Medical, Canberra, ACT, Australia), and other similar commercial antifogging liquid products. Another common measure for minimizing LLF is reducing the imbalance in temperature between the lens and abdominal cavity by warming the lens and shaft of the laparoscope in a bath of hot water. However, there is limited evidence for the effectiveness of these methods; they are often time consuming, and they can disrupt the surgical workflow [4]. New cleaning techniques that provide clear visualization and minimize interruptions to surgical workflow caused by scope contamination are therefore needed.

Microfiber cloth has been developed specifically for cleaning liquid crystal displays and other equipment. It has been reported that these cloths can clean medical equipment without using any chemicals or cleaning agents and that their cleaning effect is 8.6–10 times greater when they have been moistened [5]. The simple underlying hypothesis is that the very small‐diameter microfibers increase the area covered per area treated, maximizing the effectiveness of cleaning a laparoscopic lens. It is also possible that the shorter cleaning time decreases the time spent outside the abdominal cavity sufficiently to eliminate the decrease in scope temperature that can lead to the formation of condensation and lens fogging. In this single‐center, prospective, randomized, controlled clinical trial, we compared the effectiveness of using microfiber cloths to clean laparoscopic camera lenses with that of conventional methods using antifog solution during laparoscopic surgery for CRC.

## 2 Materials and Methods

### 2.1 Study Design and Patients

This was a single‐center, prospective, randomized study of consecutive patients diagnosed with CRC who underwent laparoscopic resection between February 2016 and August 2017 in Keio University Hospital. The eligibility criteria were as follows: (1) aged more than 18 years; (2) histologically proven CRC; (3) scheduled for laparoscopic surgery for CRC; (4) consent to randomization; and (5) signed informed consent form. The exclusion criteria were as follows: (1) scheduled for combined resection of other organs and (2) conversion to open surgery.

**FIGURE 1:**
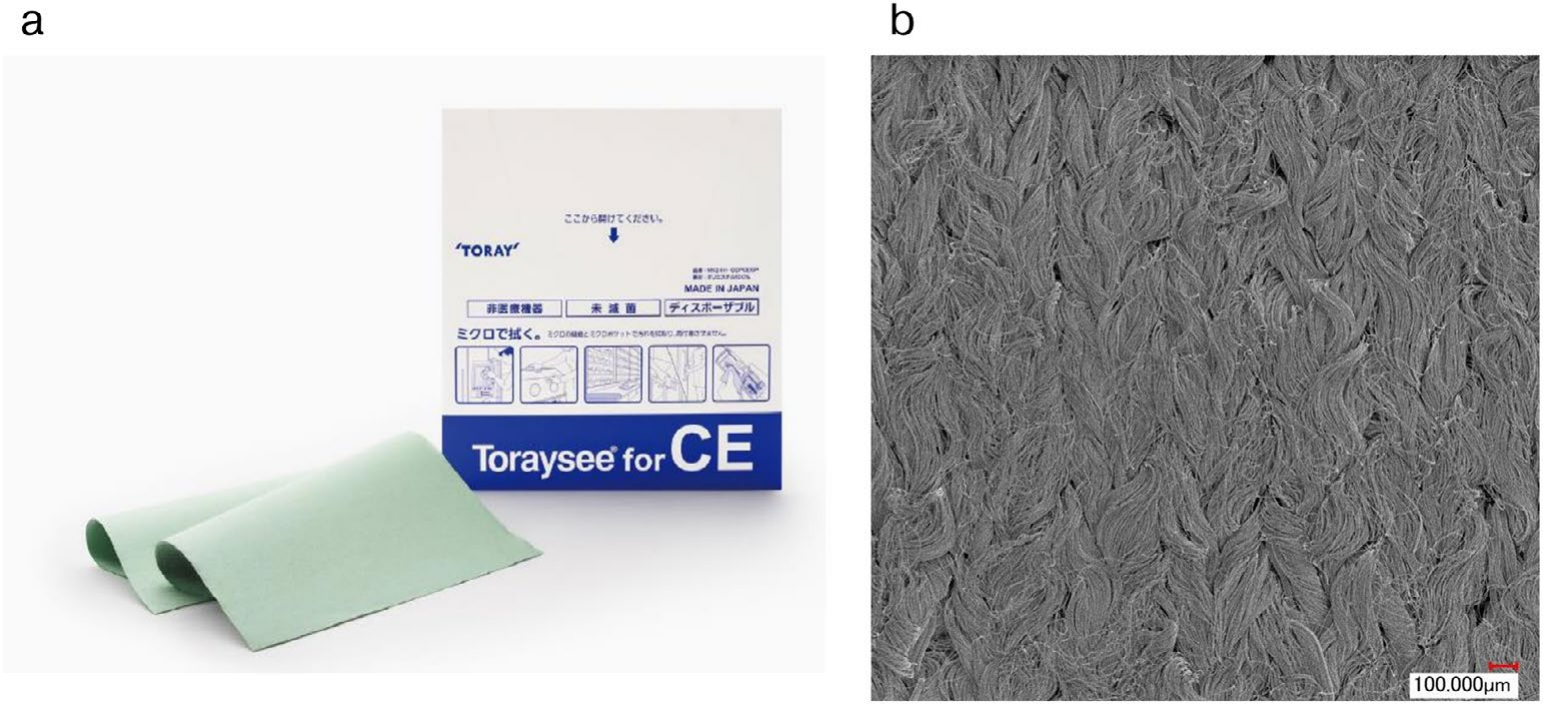
(a) A microfiber cleaning cloth (Toraysee); (b) magnified surface of a microfiber cleaning cloth.

The microfiber cleaning cloths (Toraysee for CE; Toray, Tokyo, Japan) assessed in this study are novel lens‐cleaning devices that are composed of sea‐island type composite fabric consisting of very small (2 μm diameter) polyester microfibers (Figure 1). For patients allocated to microfiber cloth, the endoscopist removed the scope, cleaned the lens with a wet microfiber cloth, and reinserted the scope. The comparable conventional lens‐cleaning method of ULTRA‐STOP (Sigmapharm, Vienna, Austria) was adopted as the control. This antifogging solution contains alcohol, surfactant, and water. Standard cleaning of a contaminated scope was performed by removing the scope, wiping the lens with wet gauze, immersion in antifog solution followed by a dry gauze blot, and reinsertion of the scope. Lens cleaning procedure was performed by laparoscopic assistant, a surgical resident with 5–7 years of clinical experience. Due to its nature, this study is open‐label trial and the surgeons were aware of which group they were allocated. During the study period, A HD, 2D flexible laparoscopic camera was consistently employed for all procedure. This study was registered with UMIN Clinical Trials Registry (http://www.umin.ac.jp/ctr/index‐j.htm) as UMIN000019356. The device and the protocol for this study were approved by the Ethics Committee of Keio University Hospital (No. 2015183). Written informed consent was obtained from all individual participants included in the study.

### 2.2 Visual Analyzing Software

**FIGURE 2:**
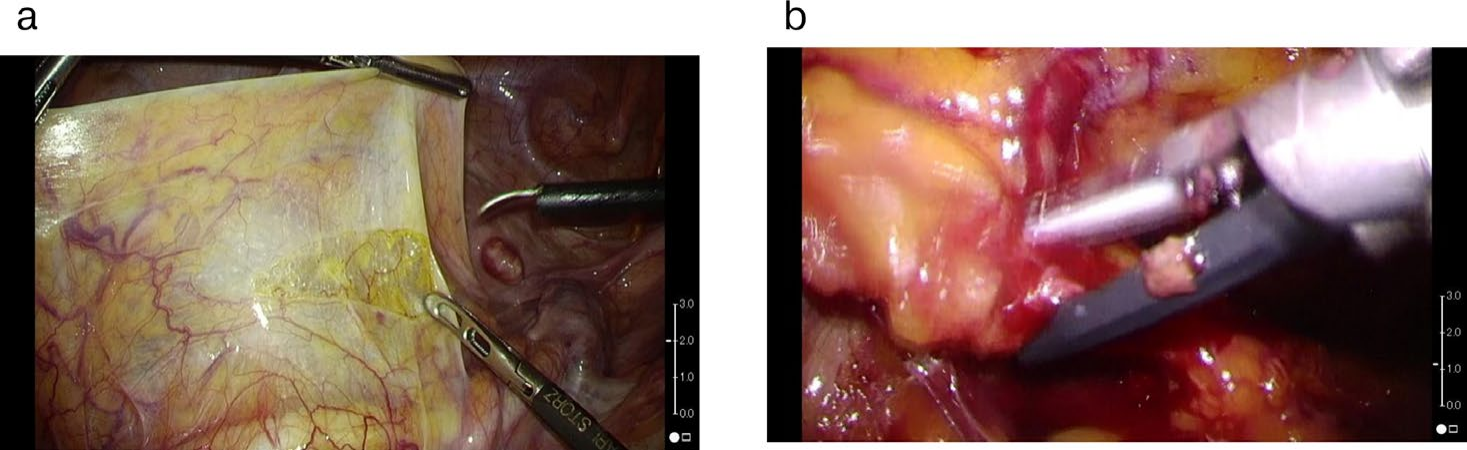
Visual representation of the scope‐cleaning scoring system. (a) score of 2 = perfectly clear view. (b) score of 1 = slightly blurred view.

We used novel visual analyzing software (LamVideoClrVal; K. K. Lamtec, Japan) to objectively assess the clarity of the laparoscopic images. This software calculates a clarity score that is the average difference in pixel values between the pixel of interest and the adjacent pixels in a still image acquired from a video of laparoscopic surgery. The clarity score is lower when the still image is cloudy or blurry and higher when the still image is sharp and not blurry. The image is generally considered clear when the clarity score is between 1.6 and 1.8 [6]. LamVideoClrVal was used to calculate the clarity scores, which varied from 0 (*completely obscure*) to 2 (*perfectly clear*), every one‐third of a second throughout each procedure (Figure 2). In this study, images from before and after cleaning the laparoscopic lens were extracted and the differences in clarity scores selected as a measure of effectiveness.

### 2.3 Outcomes of Interest

The primary outcome was mean time spent cleaning laparoscopic lens (MTCL) on each occasion during a laparoscopic CRC surgery. The secondary outcomes were the number of times the laparoscopic lens required cleaning (NTCL) per procedure and total time spent cleaning laparoscopic lenses (TTCL) per procedure. Two reviewers were involved in the analysis of the videos of the laparoscopic procedure. Time taken to remove the laparoscopic tip, clean it, reintroduce it, and place it was recorded as the time spent on each occasion of cleaning the laparoscopic lens. The number of times the laparoscope was cleaned during each procedure was also recorded. In both groups, regardless of the number of times the lens cleaning was repeated, the interval from removal to reinsertion of the scope with archiving clear visual field was counted as a single cleaning event, and the duration was recorded as time spent cleaning laparoscopic lens per one time. If there was a mismatch between reviewers, they discussed it and reached consensus. To objectively analyze the clarity of the laparoscopic view, we adopted the novel visual analysis software LamVideoClrVal. For this purpose, the differences in clarity scores between before and after each occasion of cleaning, using either the microfiber cloth or the standard (control) procedure were recorded.

### 2.4 Randomization

This was an open‐label, randomized, controlled clinical trial in patients undergoing laparoscopic CRC surgery. The required sample size was calculated using the primary outcome of MTCL. Results of retrospective preliminary data on 16 cases indicated that a difference of approximately 7 s was expected. Based on the assumptions that *α* = 0.05 (two‐sided), power = 0.90, and the ratio of control to experimental patients = 1, we calculated that a statistically significant difference of 7 s would require more than 24 patients in each study arm. We therefore set the sample size of each arm at 30 cases. Randomization was performed using a random number generator in Microsoft Excel on entry into the trial, patients being randomized to a control group (standard scope cleaning maneuvers) or microfiber cloth group (scope cleaning with a moistened microfiber cloth). All patients gave written consent to enrollment in the study and were notified after the procedure (before discharge) which arm they had been randomized to.

### 2.5 Data Collection

Relevant patient characteristics (age, sex, body mass index, history of prior abdominal surgery, American Society of Anesthesiology Classification, tumor location, and tumor stage) were collected at the time of enrollment. Surgical details, including type of procedure, mean operation time, estimated blood loss, laparoscopic time, and intraoperative complications were collected at the time of the procedure. Intraoperative and postoperative complications and mean length of stay were recorded at the time of discharge. All adverse events after surgery were recorded and graded using the Clavien–Dindo grading system [7].

### 2.6 Statistics

Between‐group comparisons of continuous data were performed using the *t‐*test or Wilcoxon rank sum test, depending on normality. Comparison of categorical data was performed using the χ^2^ or conditional binomial exact test, depending on group numbers. *p* < 0.05 were considered to denote statistical significance. Statistical analysis was performed using Stata/SE 12.1 for Mac (Stata Corporation, College Station, TX, USA) and LamVideoClrVal (K. K. Lamtec, Japan).

## 3 Results

### 3.1 Baseline Patient Characteristics

**FIGURE 3:**
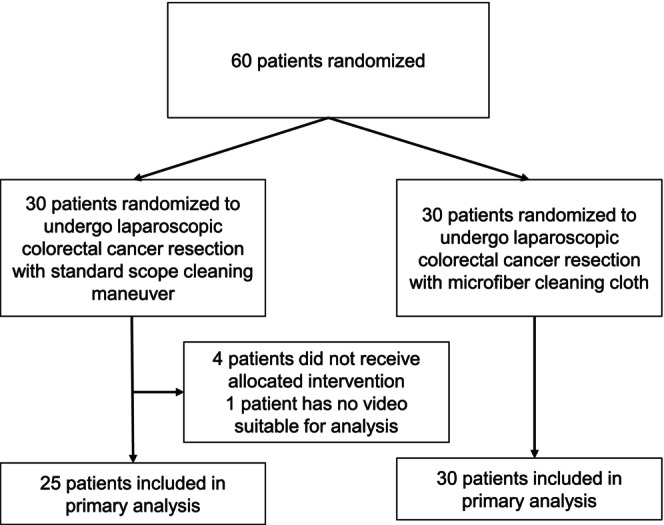
Flow diagram of patients in the study.

A flow diagram of this study is shown as Figure 3 and the patients' clinical characteristics are presented in Table 1. Between March 2016 and August 2017, 60 patients consented to participation, with 30 patients being randomized into each arm of the study. Five patients in the control group were excluded from the analysis (four did not require any laparoscopic lens cleaning during their procedures and one had no analyzable video recording). Baseline patient characteristics did not differ significantly between the study groups.

**TABLE 1:** Baseline characteristics of patients.

			Control	Microfiber cloth	
Characteristics		Total *N* = 55	*N* = 25	*N* = 30	*p*
Age (median [IQR], year)		72 (44–85)	72 (58–81)	74 (44–81)	0.324
Sex	Male	34	17	17	
	Female	21	8	13	0.426
Body mass index	Median (IQR)	21.70 (17.60–26.27)	22.15 (18.57–24.63)	20.87 (17.82–24.18)	0.340
History of prior abdominal surgery	No	39	16	23	
	Yes	16	9	7	0.303
ASA‐PS	1	22	10	12	
	2	30	14	16	
	3	3	1	2	
	4	0	0	0	
	5	0	0	0	
	6	0	0	0	0.907
Tumor location	Right	33	12	21	
	Left	17	11	6	
	Rectum	5	2	3	0.157
Tumor stage	0	2	1	1	
	l	24	7	17	
	ll	13	6	7	
	lll	15	10	5	
	lV	1	1	0	0.146

Abbreviation: ASA‐PS, American Society of Anesthesiologists‐physical status.

### 3.2 Outcomes

Outcomes of interest are shown in Table 2. At an average of 16.12 ± 4.63 s, the MTCL was significantly shorter in the microfiber cloth group than in the control group (21.04 ± 4.83 s; *p* < 0.01). Despite this difference in MTCL, the NTCL was similar in the microfiber cloth and control groups (8.5, 6–12 and 8, 5–12; *p* = 0.819). Therefore, the TTCL was significantly shorter in the microfiber cloth group (*p* < 0.01). TTCL comprised 0.89% of the operating time in the microfiber group and 1.18% of operating time in the control group.

**TABLE 2:** Outcomes and surgical details.

		Total *N* = 55	Control (*N* = 25)	Microfiber cloth (*N* = 30)	*p*
Outcomes of interest					
MTCL (mean ± SD, s)		18.36 ± 5.29	21.04 ± 4.83	16.12 ± 4.63	> 0.001
TTCL (median, IQR, s)		141 (81–227)	177 (120–237)	135 (78–212)	> 0.001
NTCL (median, IQR)		8 (6–12)	8 (5–12)	8.5 (6–12)	0.819
The difference of clarity score (median, IQR)		0.151 (0.030–0.253)	0.135 (0.030–0.250)	0.164 (0.030–0.260)	0.276
Surgical details					
Type of procedure					
Ileocecal resection		10	5	5	
Right hemicolectomy		20	13	7	
Transverse colectomy		2	2	0	
Left hemicolectomy		3	2	1	
Sigmoidcolectomy		12	3	9	
Anterior resection		5	3	2	
Abdominal perineal resection		2	1	1	
Partial resection		1	1	0	0.338
Median operation time (median, IQR, min)		251 (224–306)	249 (224–286)	252 (227–312)	0.697
Estimated blood loss (median, IQR, mL)		10 (10–50)	10 (10–50)	10 (10–50)	0.761
Intraoperative complication (no. of patients)		1	1	0	0.269
Postoperative complication (no. of patients)		10	4	6	0.702
Clavien–Dindo classification (no. of patients)					
1			1	1	
2			1	2	
3			1	3	
4			1	0	
Median length of stay (median, IQR, days)		13 (11–19)	15 (12–20)	12 (11–19)	0.254

Abbreviations: MTCL, mean time spent cleansing laparoscopic lens; NTCL, number of times the laparoscopic lens required cleaning; TTCL, total time spent cleansing laparoscopic lenses.

### 3.3 Analysis of Clarity by Visual Analyzing Software

**FIGURE 4:**
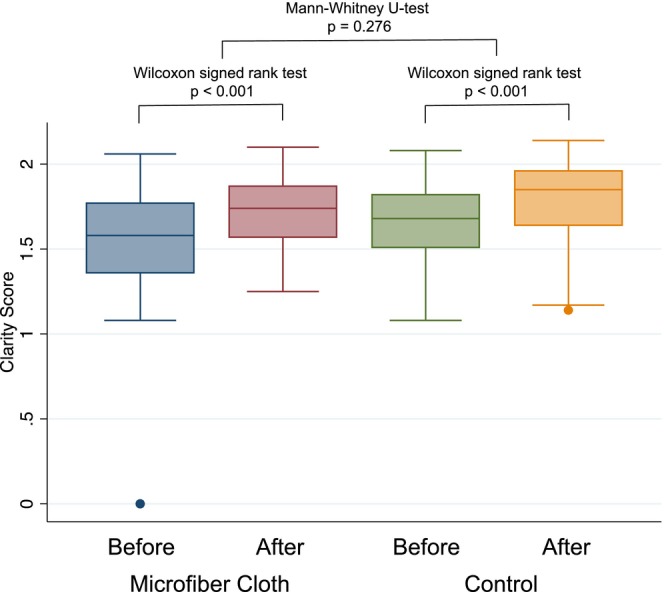
Differences in clarity scores between before and after occasions of cleaning.

The results of the clarity analysis are shown in Figure 4. The median clarity scores in the microfiber group before and after cleaning were 1.58 (1.36–1.77) and 1.74 (1.57–1.87), respectively, whereas in the control group, the median clarity scores before and after cleaning were 1.68 (1.51–1.82) and 1.85 (1.64–1.96), respectively. Significant improvements in clarity scores after cleaning were observed in both groups (*p* < 0.001). Despite the shorter time spent cleaning laparoscopic lenses, the median differences in clarity scores between before and after cleaning were 0.164 in the microfiber cloth group compared with 0.135 in the control group; this difference is not statistically significant. The mean operation time and estimated blood loss were similar in the two groups.

### 3.4 Surgical Outcomes

Other surgical outcomes are also shown in Table 2. There were no differences in intraoperative and postoperative complications between the groups (*p* = 0.269 and *p* = 0.702, respectively). Additionally, at 15 and 12 days, respectively, the median lengths of stay were similar for the microfiber cloth and control groups (*p* = 0.254).

## 4 Discussion

With the aim of improving the surgical view during laparoscopic CRC surgery, in this prospective, randomized clinical trial we compared the efficacy of a microfiber cleaning cloth with that of a conventional lens‐cleaning method. We found that microfiber cleaning cloths have several advantages. First, microfiber cleaning cloths shortened the MTCL by approximately 5 s compared with the control metho; this difference is significant. Second, despite its shorter MTCL, the effectiveness of cleaning by the microfiber cloth, as judged by NTCL and the clarity of laparoscopic images achieved, did not differ significantly from that achieved by conventional lens cleaning. Additionally, we determined the effectiveness of cleaning objectively by using novel visual analyzing software. Taken together, the significantly shorter MTCL, equivalent NTCL, and clarity after cleansing demonstrated the superiority of the microfiber cleaning cloth compared to the conventional method.

Although the degree of shortening of the MTCL achieved with Toraysee does not sound substantial, in practice it is critical to clean the laparoscopic lens immediately when optimal vision is needed. One of the strengths of the current study is the uniformity of the surgical procedure. On the basis of the findings of previously published studies, we suspect that time spent cleaning lenses, the number of times intraoperative cleaning is required, and the threshold for lens cleaning vary between surgical specialties and procedures. We therefore eliminated these sources of variation and bias in laparoscopic lens cleaning by studying only patients undergoing elective laparoscopic CRC surgery [4, 8]. Additionally, the MTCL is a good indicator relatively independent from surgical specialties and procedures. The shortened MTCL achieved using Toraysee can be expected across different surgical fields and procedures. Overall, this study provided evidence that the use of microfiber cleaning cloths is associated with shortening of the operative time and improvement in the safety of laparoscopic CRC surgery. The usefulness of Toraysee should be evaluated in different surgical fields and procedures.

A number of different means of cleaning laparoscopic lenses have previously been reported [8]. Lawrentschuk et al. divided means of reducing LLF into four broad categories of (1) means of warming the laparoscope, (2) antifogging solutions, (3) modification of the laparoscope, and (4) miscellaneous methods [3]. Most of these methods aim either to prevent condensation or directly clean contaminants such as blood, fat, or debris off the surface of the laparoscopic lens. Because minor contamination of the laparoscopic lens is associated with operative delays and increasing intraoperative bleeding, there has been an expectation that improvements in laparoscopic lenses might lead to shorter operative times and surgical safety [4, 9]. However, a recent systematic review found no difference in clinical outcomes between intervention and control groups [10]. One possible explanation for this is that withdrawal of the laparoscope and use of a cleaning solution cool the laparoscope, prejudicing the efficacy of anti‐LLF methods involving preheating. It is also plausible that the antifogging efficacy of surfactants dissipates over time as a result of laparoscope withdrawal and manual cleaning of debris and/or blood off the lens, which may remove and/or impair binding of surfactant compounds. Using a microfiber cleaning cloth is relatively easy and simple. However, in this study, we demonstrated that microfiber cleaning cloths are not significantly more effective than a conventional method of cleaning laparoscopic lenses. Additionally, microfiber cleaning cloths have the advantages of technical simplicity and cost‐effectiveness.

This prospective trial demonstrated that microfiber cleaning cloths are as effective as a conventional lens‐cleaning method. The Centers for Disease Control and Prevention guidelines emphasize the superiority of microfiber material compared with gauze cleaning for removal of microorganisms [11]. Additionally, water‐jet machining creates many gaps (micro pockets) within microfiber cleaning cloths. These micro pockets shovel soils and microorganisms onto liquid crystal displays, enhancing the effectiveness with which microfiber cleaning cloths clean medical equipment [5]. However, to the best of our knowledge, their usefulness for removing fats and liquids from the abdominal cavity during laparoscopic surgery has not previously been evaluated. The results of this study suggest that microfiber cleaning cloths absorb contaminants on laparoscopic lenses into their micro pockets and can therefore reduce the MTCL and improve visual clarity. It also should be noted that their usefulness in laparoscopic CRC surgery remains unclear. By improving surgical workflow during laparoscopic surgery, microfiber cleaning cloths may confer more safety and effectiveness than conventional cleaning methods during laparoscopic CRC surgery.

A number of novel interventions designed to achieve optimal visual clarity in laparoscopic surgery have recently been introduced. Bendifallah et al. conducted a single‐center, randomized, prospective study, and reported the usefulness of the FloShield Air System, which provides “a continuous flow of dry CO_2_ gas over the tip of the scope, instantly defogging it while continuously shielding it from condensation, debris and smoke” (http://floshield.com/) [12]. Another randomized controlled trial has reported a newly developed laparoscopic lens‐cleaning device with on‐demand CO_2_ and a saline lens wash that can be attached to a laparoscope (https://www.ciphersurgical.com) [13]. However, benefits of these technological advances in terms of reduction in need to remove the scope and clinical usefulness have not yet been demonstrated. Less costly interventions have also been reported. The LacrimaSurg is a device that is attached to the laparoscopic camera and continuously irrigates the surgical optics lens with a crystalloid fluid [14]. Endo Clear (Virtual Ports Ltd., Misgav, Israel) is another low‐tech lens‐cleaning device that can be attached to the internal abdominal wall and may reduce the need to remove the scope [8]. As more diverse interventions to improve the visual field of laparoscopic cameras are reported, low‐cost interventions may be worth prioritizing because of their cost‐effectiveness. Microfiber cleaning cloths are cost‐effective because they are cheap and can be used without any chemicals or cleaning agents. However, in this study we did not evaluate the economic aspects. Future research investigating the cost‐effectiveness of microfiber cleaning cloths is needed.

This study had some limitations. First, a number of operators were involved in this trial, and the threshold for deciding to clean the lens likely varies between operators. Second, it was not possible to blind the operators or reviewers to the allocated intervention, and there was a possibility of observational bias. Third, the outcomes of interest in this study were the duration and number of occasions of cleaning. Thus, clinically important outcomes such as operative time and complications were not evaluated. It is possible that positive effects on surgical outcomes were missed because of the relatively small sample number and limited nature of the surgical procedure. Microfiber cleaning cloths may make the surgeon less reluctant to pause to clean the lens and may also lead to shorter operative times and less workflow interruption. Future larger studies with clinical outcomes are needed.

In conclusion, the current study demonstrated that microfiber cleaning cloths are a novel cleaning method that is easy and time efficient while achieving visual quality that is equivalent to that achieved by a conventional cleaning method. Easy and shorter cleaning may lead to better surgical workflow and be associated with reduced intraoperative bleeding and complications. Further studies to assess the indirect effects associated with surgical workflow and clinical benefits are needed.

## Author Contributions

Study conception and design, acquisition of data, analysis and interpretation of data, drafting of manuscript: K. Sugiura. Study conception and design, analysis and interpretation of data, drafting of manuscript: K. Okabayashi. Study conception and design, acquisition of data, analysis and interpretation of data: K. Yamataka. Critical revision of manuscript: R. Seishima, K. Shigeta, M. Tsuruta, Y. Kitagawa.

## Disclosure

Technical support (visual analyzation) was provided for this study from Lamtec, Japan. Dr. Koji Okabayashi is an Editorial Board member of ASES Journal and a co‐author of this article. To minimize bias, they were excluded from all editorial decision‐making related to the acceptance of this article for publication.

## Conflicts of Interest

Dr. Kiyoaki Sugiura, Koji Okabayashi, and Ken Yamataka receive research funding from Toray for the present study. Dr. Koji Okabayashi received research funding from Olympus, Japan outside the present study. Dr. Ryo Seishima, Kohei Shigeta, Masashi Tsuruta, and Prof. Yuko Kitagawa declare no conflicts of interest.

## Data Availability

The data that support the findings of this study are available from the corresponding author upon reasonable request.
